# High-proportion breast milk feeding is associated with a reduction in the incidence of IVH in very preterm infants

**DOI:** 10.3389/fneur.2022.993985

**Published:** 2023-01-18

**Authors:** Zhi Zheng, Wei Shen, Li-Xia Tang, Rong Zhang, Rui Cheng, San-Nan Wang, Dong-Mei Chen, Chao Chen, Xin-Zhu Lin

**Affiliations:** ^1^Department of Neonatology, Women and Children's Hospital, School of Medicine, Xiamen University, Xiamen, Fujian, China; ^2^Xiamen Key Laboratory of Perinatal-Neonatal Infection, Women and Children's Hospital, Xiamen, Fujian, China; ^3^Department of Neonatology, Children's Hospital of Fudan University, Shanghai, China; ^4^Department of Neonatology, Children' Hospital of Nanjing Medical University, Nanjing, Jiangsu, China; ^5^Department of Neonatology, Suzhou Municipal Hospital, Suzhou, Jiangsu, China; ^6^Department of Neonatology, Quanzhou Maternity and Children's Hospital, Quanzhou, Fujian, China

**Keywords:** very preterm infants, intraventricular hemorrhage, breast milk feeding, high-proportion breast milk feeding, risk factors

## Abstract

**Objective:**

To investigate the protective effect of high-proportion breast milk feeding (>50%) on intraventricular hemorrhage (IVH) in very preterm infants (VPIs).

**Methods:**

This was a retrospective secondary analysis of a prospective multi-center study, which included 604 VPIs from six hospitals in eastern China between September 2019 and December 2020. The 604 VPIs were divided into two groups according to whether IVH occurred. High-proportion breast milk feeding was defined as breast milk accounting for 51–100% of the total feeding amount both within 7 days and throughout the hospitalization. The IVH grades and the rate of high-proportion breast milk feeding were analyzed. Furthermore, to explore the relationship between high-proportion breast milk feeding and IVH grading, the VPIs' general information, perinatal factors, growth, and nutritional status during hospitalization, and related complications were compared between the two groups.

**Results:**

High-proportion breast milk feeding was reported in 63.41% of the VPIs. Furthermore, IVH grades I–II and III–IV were noted in 39.73% (240/604) and 1.66% (10/604) of the VPIs, respectively. Univariate analysis revealed that IVH occurrence in VPIs is influenced by perinatal factors, invasive respiratory therapy, high-proportion breast milk feeding, start feeding with breast milk, the cumulative amount of early parenteral nutrition, postnatal complications, physical growth, and other factors (*P* < 0.05). After adjustments for gestational age, birth weight, and possible influencing factors through binary logistic regression analysis, the results revealed that high-proportion breast milk feeding and and start feeding with breast milk were associated with a lower total incidence of IVH. Further stratification showed that high-proportion breast milk feeding was associated with a lower incidence of grade I–II IVH. Similarly, after adjusting for the same factors, breast milk feeding >50% in the 1st week was associated with a decreased incidence of total IVH and further stratification showed that it was associated with a lower incidence of grade I–II IVH.

**Conclusion:**

High-proportion breast milk feeding and breast milk feeding more than 50% of total intake during the 1st week might be protective factors for IVH grade I–II in VPIs, which further verified the neuroprotective effect of breast milk. In clinical practice, the construction of breast milk banks should be strengthened, breast milk feeding should be encouraged in neonatal intensive care units, and efforts should be made to increase breast milk feeding rates to improve the outcomes of VPIs.

## Introduction

Very preterm infants (VPIs) have immature nervous systems and are susceptible to brain injury due to hypoxia, ischemia, infection, and other factors. Intraventricular hemorrhage (IVH) is the most common type of brain injury and is associated with death, neurodevelopmental delay, and cerebral palsy in VPIs (1–5). Studies published in 2019–2022 reported that in China, the rates of IVH grades I–II and III–IV in preterm infants with gestational age 28–31^+6^ weeks were 14.3% and 2.7–5.2%, respectively. Among preterm infants with gestational age < 28 weeks, the rates of IVH grades I–II and III–IV were 18.7–21.8% and 10.8–14.5%, respectively (6–8). The incidence of IVH grade III–IV in preterm infants with gestational age < 28 weeks has been significantly higher in China than in developed countries (5.2–7%) (9, 10). Multiple studies have demonstrated that breast milk can reduce the incidence of necrotizing enterocolitis (NEC), bronchopulmonary dysplasia (BPD), retinopathy of prematurity, and late-onset sepsis and improve neurodevelopmental outcomes at a corrected gestational age of 24 months (11–16). Long-chain polyunsaturated fatty acids and oligosaccharides in breast milk can promote brain development and myelination, improve white matter microstructure, and increase cortical thickness through inflammation reduction (17, 18). Various bioactive factors contained in breast milk, particularly colostrum, may also have protective effects for IVH in preterm infants. Exclusive breast milk feeding in preterm infants is challenging in China, but a certain percentage of breast milk feeding is achievable in the neonatal intensive care unit (NICU). Hence, this study was conducted to explore the relationship between high-proportion breast milk feeding and IVH occurrence in VPIs.

## Objectives and methods

### Research objectives

#### Patient source and grouping

This study is a retrospective secondary analysis of a multi-center prospective observational study, including 604 VPIs from a neonatology department of six tertiary hospitals in East China from September 2019 to December 2020, namely four children's hospitals, one tertiary general hospital, and one maternal and child health care hospital. For the analysis of influencing factors, the infants were divided into two groups based on whether IVH occurred.

#### Inclusion and exclusion criteria

The inclusion criteria were as follows: (1) gestational age at birth < 32 weeks, (2) hospitalization time >2 weeks, and (3) admission within 24 h after birth.

The exclusion criteria were as follows: (1) congenital malformations or genetic metabolic diseases; (2) underwent various surgical operations during the neonatal period, except for NEC; (3) hospital stay ≤ 2 weeks; (4) death during hospitalization, treatment interruption, or automatic discharge; and (5) incomplete information.

#### Ethics and clinical registration

This study was initiated and organized by the Nutrition Professional Committee of the Neonatologist Branch of the Chinese Medical Doctor Association and was registered in the Chinese Clinical Trials Registry with the registration number ChiCTR1900023418. The research protocol was approved by the Ethics Committee of Xiamen University Affiliated Women and Children's Hospital/Xiamen Maternal and Child Health Hospital (batch number KY-2019–016).

### Research methods

#### Data collection

The VPIs' general information, perinatal factors, nutritional status during hospitalization, growth, and complications were collected according to a unified questionnaire. Perinatal data (gestational age at birth, birth weight, sex, delivery mode, multiple births, small-for-gestational-age infants, prenatal hormone use, 5-min Apgar score ≤ 7, and the duration of invasive mechanical ventilation use), maternal comorbidities (gestational hypertension and gestational diabetes mellitus), nutrition and growth-related indicators during hospitalization (time to start feeding; start feeding with breast milk; the proportion of breast milk feeding >50%; accumulative doses of amino acid, fat emulsions, and calorie intake during the first week of hospitalization; maximum body weight loss; days to regain birth weight; growth velocity after regaining birth weight; and head circumference [change in Z score, ΔZ] value during hospitalization), and primary complications during hospitalization (neonatal respiratory distress syndrome [NRDS], BPD, early-onset sepsis [EOS], NEC, IVH, periventricular leukomalacia [PVL], hemodynamically significant patent ductus arteriosus [hsPDA], and extrauterine growth restriction [EUGR]) were compared, and the related influencing factors of IVH were analyzed.

#### Data quality control

The data entry personnel of each participating unit were uniformly trained, and the entered data were in strict accordance with the requirements of the research program. EpiData database was established in this study, and the data of the case report form were recorded by two persons. Each participating unit collected and uploaded the relevant data of VPIs in time and locked the database after verification and confirmation. The team leader maintained close contact with all participating units, checked the case records, and solved the possible problems in time. Considering the relatively uniform treatment regimens, this study selected data from six tertiary hospitals in the economically developed East China region for analysis.

#### Definitions, diagnostic criteria, and calculation methods of related diseases

Small for gestational age was defined as newborns whose birth weight is lower than the 10th percentile of the average birth weight of the same sex and gestational age. EUGR was defined according to the same-sex Fenton growth curve in 2013 (19) as body weight below the 10th percentile at the corrected gestational age of 36 weeks or discharge. For the clinical diagnosis and diagnostic criteria of EOS, we referred to the expert consensus published in 2019 (20). Furthermore, hsPDA was defined as PDA catheter diameter >1.5 mm, left atrial diameter/aortic diameter ≥1.4 mm, or left ventricular end-diastolic diameter/aortic diameter ≥2.1 mm, accompanied by one of the following clinical manifestations: heart murmur, tachycardia (≥160 beats/min), increased breathing, increased pulse pressure (>25 mmHg), hypotension, flushing, or cardiac dilation. For IVH diagnostic and grading criteria, we referred to Volpe's Neurology of the Newborn (21). Growth velocity after regaining birth weight (g/kg·d) was calculated as follows: GV = [1,000 × ln(Wn/W1)]/(Dn-D1), where GV is growth velocity, Wn is weight at discharge (g), W1 is the birth weight (g), Dn is the length of hospital stay (days), and D1 is days to regain birth weight (days). High-proportion breast milk feeding was defined as breast milk (consisting of the mother's breast milk and donated human milk) accounting for 51–100% of the total feeding amount both within 7 days and throughout the hospitalization. Contrarily, low-proportion breast milk feeding was defined as breast milk accounting for 0–50% of the total feeding amount both within 7 days and throughout the hospitalization, including those infants fed with formula exclusively. For the diagnosis of NRDS, BPD, NEC, and PVL, we referred to *Practical Neonatology* (fifth edition) (22).

#### Nutrition program of VPIs during hospitalization

The nutrition program of VPIs during hospitalization was based on the published guidelines and expert consensus (23–25) with appropriate adjustments. VPIs were initiated with breast milk feeding as early as possible. Maternal breast milk feeding was the first choice, and if mother's milk was unavailable, donated human milk was the second choice; the third choice was formula milk. Feeding was started within 12 h after birth for infants without congenital gastrointestinal malformations, suspected or diagnosed NEC, hemodynamic instability, or multiple organ dysfunction. Tube feeding was started after birth and was gradually transitioned to oral feeding at the corrected gestational age of 32–34 weeks. When the total breast milk feeding amount reached 50–80 mL/kg/d, breast milk was added as a fortifier. Total enteral feeding was defined as the enteral feeding amount of 150 mL/kg/d. Parenteral nutrition was started as soon as possible (12–24 h after birth) and administered through a central vein (umbilical venous catheter or peripherally inserted central catheter). The initial amino acid dose was 1.5–2 g/kg/d, and the maximum dose was 3.5–4.0 g/kg/d. The initial fat emulsion dose was 1.0 g/kg/d, which increased by 0.5–1.0 g/kg/d, and the maximum dose was 3.0 g/kg/d. The recommended initial glucose infusion dose was 4–8 mg/kg/min and was increased by 1–2 mg/kg/min per day, with a maximum dose of 11–14 mg/kg/min. When the enteral feeding amount reached 120 mL/kg/d, intravenous nutrition was stopped.

#### Protocols of monitoring IVH and PVL in VPIs

All VPIs were monitored through cranial ultrasonography at 1–3 days after birth, 7 days after birth, and every 1–2 weeks thereafter. For preterm infants with positive results of cranial ultrasonography, magnetic resonance imaging was recommended before discharge or at a corrected gestational age of 36 weeks.

### Statistical analysis

SPSS 23.0 software was used for the statistical analysis of the data. The measurement data that conform to the normal distribution are expressed as mean±standard deviation, the measurement data that do not conform to the normal distribution are expressed as the median (P25, P75), and the count data are expressed as the frequency (percentage). Continuous variables were compared between the two groups by using the *t*-test or Mann–Whitney *U* test, and categorical variables were compared using the chi-square test or Fisher's exact test. Collinearity diagnostic analysis was performed because of the possibility of multicollinearity in >50% breastfeeding during the 1st week and a high proportion of breastfeeding. Binary Logistic regression model was used to correct the possible influencing factors. Odds ratio (OR) and 95% confidence interval (CI) were used to compare the association between high proportion of breastfeeding, breastfeeding >50% in the 1st week, breastfeeding initiation with the risk of IVH and IVH with different stratification (shown in **Tables 4**, **5**). *P* < 0.05 was considered statistically significant.

## Outcomes

### General information

**Figure 1:**
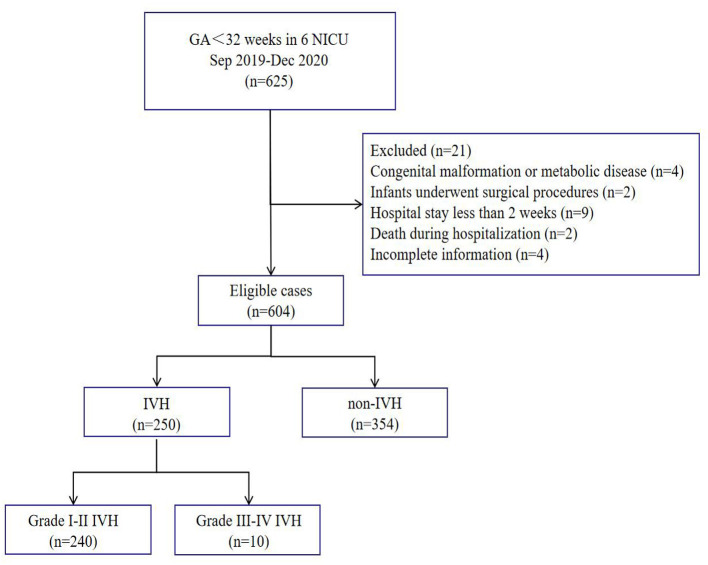
Flow chart of participant selection and categorization in the study.

During the study period, 625 VPIs met the inclusion criteria, of whom 21 were excluded according to the exclusion criteria, and 604 VPIs were finally included for the statistical analysis (Figure 1). The average gestational age at birth was 29.93 ± 1.52 weeks, and the average birth weight was 1395.00 ± 298.97 g. The gestational age at birth was < 26 weeks in 9 VPIs, 26–27^+6^ weeks in 66 VPIs, 28–29^+6^ weeks in 1,176 VPIs, and 30–31^+6^ weeks in 353 VPIs. The birth weight was < 750 g in 4 VPIs, 750–999 g in 58 VPIs, 1,000–1,249 g in 131 VPIs, 1,250–1,499 g in 173 VPIs, and >1,500 g in 238 VPIs.

The characteristics of all and high-proportion breast milk feeding VPIs are presented in Table 1. The results showed that 63.41% (383/604) VPIs received high-proportion breast milk feeding, and the rates of IVH grades I–II and III–IV among the VPIs were 39.73% (240/604) and 1.66% (10/604), respectively. Among preterm infants with a gestational age of < 28 weeks, the rates of IVH grades I–II and III–IV were 40.00% (30/75) and 5.33% (4/75), respectively. Among preterm infants with a gestational age of 28–31^+6^ weeks, the incidences of IVH grades I–II and III–IV were 39.70% (210/529) and 1.13% (6/529), respectively. The incidence of IVH grades I–II was lower in the high-proportion breast milk feeding group (χ2 = 25.38, *P* < 0.001), while the incidence of IVH grades III–IV and PVL was not associated with high-proportion breast milk feeding (*P* > 0.05). The high-proportion breast milk feeding group had a higher proportion of start feeding with breast milk (χ2 = 57.21, *P* < 0.001).

**Table 1:** Characteristics of all VPIs and high/low-proportion breast milk feeding VPIs.

**Factors**	**All VPI cases** **(*n* = 604)**	**VPI with a high-proportion of breast milk feeding** **(*n* = 383)**	**VPI with a low-proportion of breast milk feeding** **(*n* = 221)**
Gestational age ^a^ (week)	30.1 (28.9,31.1)	30.0 (28.6,31.0)	30.6 (29.6,31.4) ^*^
< 28 week, *n* (%)	75 (12.4)	57 (14.9)	18 (8.1) ^*^
28~31^+6^week, *n* (%)	529 (87.6)	326 (85.1)	203 (91.9)
Birth weight ^a^ (gram)	1397.5 (1,200, 1,600)	1,380 (1,150, 1,600)	1,400 (1,203, 1,630)
Male, *n* (%)	348 (57.6)	224 (58.5)	124 (56.1)
Start of feeding ^a^ (hour)	24 (18,38)	25 (20,36)	24 (17,48)
Start feeding with breast milk, *n* (%)	234 (38.7)	192 (50.1)	42 (19.0) ^*^
Cumulative milk volume during W1^b^ (ml/Kg)	197.3 ± 25.3	196.9 ± 25.8	198.6 ± 24.9
The incidence of IVH I-II, *n* (%)	240 (39.7)	123 (32.1)	117 (52.9) ^*^
The incidence of IVH I-II among infants with GA < 28, *n* (%)	30 (40)	18 (31.6)	12 (66.7)
The incidence of IVH I-II among infants with GA 28~31^+6^, *n* (%)	210 (39.7)	105 (27.4)	105 (51.7)
The proportion of infants developed IVH I-II within 3 days, *n* (%)	200 (83.3)	101 (82.1)	99 (84.6)
The proportion of infants developed IVH I-II within 7 days, *n* (%)	223 (92.9)	111 (90.2)	112 (95.7)
The incidence of IVH III-IV, *n* (%)	10 (1.7)	6 (1.6)	4 (1.8)
The incidence of IVH III-IV among infants with GA < 28, *n* (%)	4 (5.3)	2 (3.5)	2 (11.1)
The incidence of IVH III-IV among infants with GA 28~31^+6^, *n* (%)	6 (1.1)	4 (1.2)	2 (1.0)
The proportion of infants developed IVH III-IV within 3 days, *n* (%)	8 (80)	5 (83.3)	3 (75)
The proportion of infants developed IVH III-IV within 7 days, *n* (%)	10 (100)	6 (100)	4 (100)
The incidence of PVL, *n* (%)	64 (10.6)	45 (11.7)	19 (8.6)

a Expressed as median (P25, P7.5),

b Expressed as mean ± standard deviation (X ± SD).

* Represents the comparison of the factor between the high and low proportion of breast milk feeding groups, *P* < 0.05.

GA, geatational age; VPI, very premature infants; W1, the 1st week; IVH, intraventricular hemorrhage; PVL, periventricular leukomalacia.

The infants were divided into two groups according to IVH occurrence, and the perinatal characteristics of the VPIs between the two groups were compared (Table 2). The IVH group had lower rates of prenatal hormone use, cesarean section, and hsPDA incidence and higher incidence of EOS and longer invasive respiratory treatment time; the differences were statistically significant (*P* < 0.05).

**Table 2:** Perinatal characteristics of VPIs in the two groups.

	**IVH (*n* = 250)**	**Non-IVH (*n* = 354)**	**χ2/t/Z**	***P***
Gestational age ^a^ (week)	30.1 (29.0, 31.1)	30.3 (28.9, 31.1)	0.521	0.602
groups, *n* (%)			0.660	0.883
< 26	4 (1.6)	5 (1.4)		
26–27^+6^	26 (10.4)	40 (11.3)		
28–29^+6^	77 (30.8)	99 (28.0)		
30–31^+6^	143 (57.2)	210 (59.3)		
Birth weight ^a^ (gram)	1390.0 (1200.0, 1560.0)	1400.0 (1198.8,1632.5)	0.830	0.406
Groups, *n* (%)			-	0.666
< 750	3 (1.2)	1 (0.3)		
750–999	21 (8.4)	37 (10.5)		
1,000–1,249	55 (22.0)	76 (21.5)		
1,250–1,499	73 (29.2)	100 (28.2)		
≥1,500	98 (39.2)	140 (39.5)		
Head circumference at birth ^a^ (cm)	27.6 (26.0, 29.0)	27.5 (26.0, 29.0)	0.871	0.384
Male, *n* (%)	145 (58.0)	203 (57.3)	0.026	0.872
Prenatal steroids, *n* (%)	209 (83.6)	317 (89.5)	4.609	0.032
Cesarean delivery, *n* (%)	120 (48.0)	211 (59.6)	7.966	0.005
Multiple pregnancies, *n* (%)	78 (31.2)	102 (28.8)	0.399	0.528
SGA, *n* (%)	22 (8.8)	18 (5.1)	3.271	0.071
Apgar ≤ 7 (5 min), *n* (%)	28 (11.2)	40 (11.3)	0.001	0.970
Maternal gestational diabetes, *n* (%)	48 (19.2)	70 (19.8)	0.031	0.861
Maternal gestational hypertension, *n* (%)	36 (14.4)	52 (14.7)	0.010	0.921
RDS, *n* (%)	178 (71.2)	241 (68.1)	0.672	0.412
Early-onset sepsis, *n* (%)	44 (17.6)	20 (5.6)	22.088	< 0.001
hsPDA, *n* (%)	82 (32.8)	164 (46.3)	11.107	0.001
Invasive MV time (day)	1 (0, 3)	0 (0, 2)	4.013	< 0.001
Endotracheal intubation within the 1st week, *n* (%)	190(76)	247(69.8)	2.839	0.092
Outborn and transffered, *n* (%)	181(72.4)	266(75.1)	0.572	0.449

a Expressed as median (P25, P75).

VPI, very premature infants; IVH, intraventricular hemorrhage; SGA, small for gestational age; RDS, respiratory distress syndrome; hsPDA, hemodynamically significant patent ductus arteriosus; MV, mechanical ventilation.

### Analysis of the influencing factors of IVH in VPIs

#### Analysis of nutritional status, growth, and complications of preterm infants between the two groups

The patients were divided into two groups according to whether IVH occurred. The nutritional status, growth, and complications of premature infants were compared between the two groups. The results are presented in Table 3. The results suggested that VPIs with IVH had a lower probability of receiving high-proportion breast milk feeding, a lower incidence of start feeding with breast milk, less infants receiving breast milk feeding more than 50% of total intake during the 1st week, lower amino acid accumulation and calorie accumulation in the 1st week, and lower head circumference (ΔZ value) during hospitalization. Furthermore, in the 1st week, the cumulative amount of fat was higher, the weight loss was larger, and the incidence of NEC and EUGR at discharge was higher in the IVH group than in the non-IVH group. The difference was statistically significant (*P* < 0.05).

**Table 3:** Comparison of nutritional status, growth, and complications of preterm infants between the two groups.

	**IVH (*n* = 250)**	**Non-IVH (*n* = 354)**	**χ2/t/Z**	***P***
The start of feeding ^a^ (hour)	24 (15.0, 47.3)	24 (20.0, 35.3)	0.403	0.687
Start feeding with breast milk, *n* (%)	77 (30.8)	157 (44.4)	11.336	0.001
Breast milk feeding more than 50% of total intake during W1, *n* (%)	136 (54.4)	263 (74.3)	25.864	< 0.001
High proportion breast milk feeding, n (%)	128 (51.2)	255 (72.0)	27.411	< 0.001
Cumulative milk volume during W1^b^ (ml/Kg)	193.9 ± 31.8	197.5 ± 26.9	1.416	0.157
Cumulative dose of amino acid during W1 ^a^ (g/Kg)	16 (13.7, 17.8)	17.9 (15.4, 19.7)	6.447	< 0.001
Cumulative dose of fat emulsions during W1 ^a^ (g/Kg)	12.4 (10.5, 13.9)	11 (8.9, 14.2)	3.330	0.001
Cumulative calories during W1 ^a^ (kcal/Kg)	475.5 (427.7, 520.6)	506.9 (445.7, 550.9)	4.548	< 0.001
The maximum weight loss ^a^ (%)	8 (4.8, 10.9)	6.2 (3.9, 9.0)	3.921	< 0.001
Days to regain birth weight ^a^ (d)	10 (8, 13)	10 (8, 12)	1.332	0.183
Weight growth velocity ^a^ (g/kg.d)	13.8 (12.3, 15.8)	14.4 (12.5, 16.1)	1.584	0.113
BPD, *n* (%)	112 (44.8)	131 (37.0)	3.702	0.054
NEC, *n* (%)	38 (15.2)	24 (6.8)	11.278	0.001
EUGR, *n* (%)	125 (50.0)	124 (35.0)	13.555	< 0.001
ΔZ of head circumference during hospitalization ^a^	0.8 (0.3, 1.3)	1.1 (0.4, 1.7)	4.236	< 0.001

a Expressed as median (P25, P75).

IVH, intraventricular hemorrhage; W1, the 1st week after birth; BPD, bronchopulmonary dysplasia; NEC, necrotizing enterocolitis; EUGR, extrauterine growth retardation; ΔZ, change in Z score, W1, the 1st week.

#### Analysis of high proportion-breast milk feeding, start feeding with breast milk, and IVH risk in VPIs

Since the data of breast milk feeding >50% in the 1st week and high proportion breast milk feeding were similar, we carried out collinearity diagnostic analysis, and the results showed that Tolerance was 0.171, < 0.2; The variance inflation factor was 5.858, >5, indicating collinearity among variables. Hence, binary logistic regression analysis was performed to compare breast milk feeding >50% in the 1st week and high proportion breast milk feeding with other significant univariate factors.

After adjustments for gestational age, birth weight, and possible influencing factors by using binary logistic regression analysis, the results suggested that high-proportion breast milk feeding and and start feeding with breast milk were associated with a lower total incidence of IVH, with the adjusted OR (95% CI) values being 0.492 (0.327, 0.739) and 0.513 (0.340, 0.773), respectively. Further stratification showed that high-proportion breast milk feeding was associated with a lower incidence of grade I–II IVH, with adjusted OR (95% CI) value of 0.547 (0.359, 0.832). Similarly, after adjusting for the same factors, breast milk feeding >50% in the 1st week was associated with a decreased incidence of total IVH, with an aOR and 95% CI of 0.547 (0.367, 0.815). Further stratification showed that breastfeeding >50% in the 1st week was associated with a lower incidence of grade I–II IVH, with an aOR and 95% CI of 0.562 (0.379, 0.835) (shown in Tables 4, 5). Adjusted factors included gestational age at birth, birth weight, cesarean section, prenatal steroid, SGA, start of feeding, early onset sepsis, hsPDA, endotracheal intubation within the 1st week, outborn and transfferd, duration of invasive ventilator, cumulative amount of amino acids, fat emulsion and calories in the 1st week, and weight loss.

**Table 4:** Analysis of the association of high-proportion breast milk feeding and start breast milk feeding with IVH (include IVH, I-II IVH, III-IV IVH) risk in VPIs.

	**IVH**			
	**Crude OR**	**95% CI**	**Adjusted OR**	**95% CI**
Start feeding with breast milk	0.558	0.397–0.785	0.513	0.340–0.773
High proportion breast milk feeding	0.407	0.290–0.572	0.492	0.327–0.739
	I-II IVH			
	Crude OR	95% CI	Adjusted OR	95% CI
Start feeding with breast milk	0.534	0.379–0.754	-	-
High proportion breast milk feeding	0.408	0.290–0.574	0.547	0.359–0.832
	III-IV IVH			
	Crude OR	95% CI	Adjusted OR	95% CI
Start feeding with breast milk	1.594	0.456–5.566	NA	NA
High proportion breast milk feeding	0.863	0.241–3.093	NA	NA

VPI, very premature infants; IVH, intraventricular hemorrhage.

Adjusted factors included gestational age at birth, birth weight, cesarean section, prenatal steroid, SGA, start of feeding, early onset sepsis, hsPDA, endotracheal intubation within the 1st week, outborn and transfferd, duration of invasive ventilator, cumulative amount of amino acids, fat emulsion and calories in the 1st week, and weight loss.

**Table 5:** Analysis of the association of breast milk feeding more than 50% of total intake during the 1st week and start breast milk feeding with IVH (include IVH, I-II IVH, III-IV IVH) risk in VPIs.

	**IVH**			
	**Crude OR**	**95% CI**	**Adjusted OR**	**95% CI**
Start feeding with breast milk	0.558	0.397–0.785	-	-
Breast milk feeding more than 50% of total intake during W1	0.413	0.292–0.583	0.547	0.367–0.815
	I-II IVH			
	Crude OR	95% CI	Adjusted OR	95% CI
Start feeding with breast milk	0.534	0.379–0.754	-	-
Breast milk feeding more than 50% of total intake during W1	0.431	0.305–0.608	0.562	0.379–0.835
	III-IV IVH			
	Crude OR	95% CI	Adjusted OR	95% CI
Start feeding with breast milk	1.594	0.456–5.566	NA	NA
Breast milk feeding more than 50% of total intake during W1	0.508	0.145–1.774	NA	NA

VPI, very premature infants; IVH, intraventricular hemorrhage; W1, the 1st week.

Adjusted factors were the same as in Table 4.

## Discussion

The subependymal germinal stroma contains a rich capillary bed that lacks tight junctions and is irregular in shape. These capillary beds are susceptible to ischemia and hypoxia, reperfusion, infection, and other factors, leading to hemodynamic disturbances. This results in the hemorrhage of the germinal matrix and rupture into the ventricular system, eventually leading to IVH occurrence. IVH is the most common type of brain injury and is closely associated with long-term adverse neurological outcomes (1–5). In our study, the rates of IVH grades I–II and III–IV were 39.73 and 1.66%, respectively. In this study, the incidence of grade I-II IVH was generally high, mainly because most of the VPI in this study came from children's hospital and neonatal rescue center. 74.01% of the children experienced extrauterine transport within 24 h after birth, and the rate of endotracheal intubation within the 1st week was 72.35%, which may lead to the increased incidence of IVH. The sample size of preterm infants < 28 weeks was small in this study, if the sample size is further expanded, the differences in the incidence of grade I-II IVH among different gestational age groups may be more significant and the data results will be more representative.

IVH is currently considered a multifactorial disease, where gestational age at birth, prenatal hormone use, cesarean section, early-onset infection, invasive respiratory therapy, and resuscitation of neonatal asphyxia are the main factors influencing IVH (10, 26, 27). A study by Carome et al. (28) demonstrated that for very low birth weight infants, early initiation and continuation of exclusive breast milk feeding until the corrected gestational age of 34 weeks was associated with a low incidence of IVH grade III–IV and PVL. Torres-Muñoz et al. (29) found that preterm infants who achieved total enteral nutrition with exclusive breast milk feeding within 7 days of life had an 85% lower incidence of IVH than those who reached the same target after 8 days of life. Exclusive breast milk feeding has been speculated to exert a protective effect on IVH in preterm infants, and therefore, this study further hypothesized and explored this speculation.

Not only have clinical studies reported on the protective effect of exclusive breast milk feeding on IVH in preterm infants (28–30) but also animal studies have reported that feeding colostrum to fetal mice early after birth can reduce apoptosis after IVH (31). Therefore, bioactive components in colostrum may cross the intestinal barrier and enter the bloodstream to exert important immune-regulatory and anti-inflammatory effects. Breast milk can help reduce the incidence of IVH, and the possible mechanisms are as follows. One, transforming growth factor β (TGF-β) is the most abundant cytokine family in breast milk, and its content in colostrum is much higher than that in mature milk (32, 33). In animal models, TGF-β plays an important role in the stabilization of the blood-brain barrier, and the disruption of the TGF-β pathway could induce IVH occurrence (34). In preterm infants, TGF-β is hypothesized to exert protective effects by reducing anticoagulant factor levels in the brain and stimulating pericyte production, providing structural support for the germinal matrix vasculature and promoting maturation (35). Two, epidermal growth factor (EGF) is a growth factor contained in breast milk that promotes cell proliferation and maturation (32, 36). EGF binds to the EGF receptor in the brain, promotes astrocyte differentiation, oligodendrocyte progenitor cell proliferation, and neuron development, which further improves the blood–brain barrier and promotes gray and white matter maturation (37–39). Therefore, EGF is considered to play an important role in IVH prevention and recovery. Three microRNAs (miRNAs) are small non-coding RNAs that are present in breast milk and can be absorbed through intestinal wall endocytosis and play a role in posttranscriptional gene regulation and immune protection (40–42). Furthermore, miRNAs can promote the recovery of hypoxic-ischemic encephalopathy and maintain the stability of the blood–brain barrier after intracerebral hemorrhage (43, 44), which may be another potential mechanism by which breast milk prevents IVH occurrence.

The exclusive breast milk feeding rate of VPIs in NICUs in developed countries is high (>90%), but it is still difficult to implement exclusive breast milk feeding for VPIs in China. Insufficient breast milk secretion at the time of mother–infant separation, contraindications to breast milk feeding in infants, and the general lack of breast milk banks, donated breast milk, and human milk fortifiers all affect the rate of exclusive breast milk feeding in China. China currently lacks large-sample-size multi-center studies on breast milk feeding rates in VPIs. The Pediatric Hospital of Fudan University reported that the breast milk feeding rate of very low birth weight infants during hospitalization reached 84% after continuous quality improvement. Our study observed high-proportion breast milk feeding in 63.41% of the VPIs, which indicates that high-proportion breast milk feeding in China is achievable. However, whether high-proportion breast milk feeding has neuroprotective effects on VPIs is unclear.

This study analyzed the relationship between high-proportion breast milk feeding and the occurrence of different grades of IVH and PVL in VPI and found that the rate of IVH grade I–II was decreased in the high-proportion breast milk feeding group (32.1 vs. 52.9%, *P* < 0.001), whereas no significant difference was observed in the rate of IVH grade III–IV and PVL between the high-/low-ratio breast milk feeding groups. After adjustment for gestational age, birth weight, and possible influencing factors through a binary logistic regression analysis, the results showed that high-proportion breast milk feeding and breast milk feeding more than 50% of total intake during the 1st week were associated with a lower incidence of IVH, and further stratification showed that these two factors were also associated with a lower incidence of IVH grade I–II. That is, high-proportion breast milk feeding in VPIs can reduce the occurrence of IVH grade I–II by 45.3%. Therefore, this study showed that high-proportion breast milk feeding and breast milk feeding more than 50% of total intake during the 1st week might be protective factors for IVH occurrence in VPIs and mainly has a protective effect on IVH grade I–II, whereas no statistical difference was observed in IVH grade III–IV, which is inconsistent with a study reported by Torres (29). The possible reasons are as follows. First, IVH is a multifactor disease. The main influencing factors are gestational age at birth, use of prenatal hormones, and invasive respiratory therapy. Breast milk may be a neuroprotective component. In this study, the aforementioned risk factors were included in the binary logistic regression analysis. Therefore, we believe that for IVH grades III–IV, the high-risk factors may be more complex and severe, and the neuroprotective effect of breast milk feeding may be diluted among many influencing factors. Conversely, for IVH grades I–II, the high-risk factors were few and less complex and severe, and high-proportion breast milk feeding had a neuroprotective effect. In addition, as breast milk feeding has a protective effect on both prevention and repair of IVH, some mild cases of IVH have effectively improved. Thus, high-proportion breast milk feeding may reduce the likelihood of IVH progression from grade I–II to grade III–IV among some infants. Second, the neuroprotective effect of breast milk is time-and dose-dependent. This study considered data of only high-proportion breast milk feeding, which may not completely reflect the neuroprotective effect of breast milk. Third, this study had few patients with IVH grade III–IV, and effective statistical analysis could not be carried out. It is necessary to include a larger sample size for further research.

The data for this study were obtained from a multi-center study and had good representativeness. This study has several limitations. First, this study was not a prospective randomized trial, and certain differences in baseline characteristics exist between the two groups; therefore, our results must be interpreted with caution. Second, this study lacks data records on hypothermia in delivery room, hypotension and vasoactive drug use in preterm infants, which have clear effects on IVH; this may cause bias in the results. Lastly, this study lacks follow-up assessments of neurodevelopmental outcomes or cognitive function after VPI discharge. Therefore, prospective randomized controlled studies are needed with larger sample sizes and follow-up data analysis.

## Conclusions

This study concludes that high-proportion breast milk feeding and breast milk feeding more than 50% of total intake during the 1st week might be protective factors for IVH grade I–II in VPIs, which further verifies the protective effect of breast milk. Follow-up studies are needed to confirm and further clarify the protective mechanism. Therefore, the construction of breast milk banks should be strengthened, breast milk feeding should be encouraged in NICUs, and efforts should be made to increase breast milk feeding rates to improve the outcomes of VPIs.

## Data availability statement

The raw data supporting the conclusions of this article will be made available by the authors, without undue reservation.

## Ethics statement

The studies involving human participants were reviewed and approved by Women and Children's Hospital, School of Medicine, Xiamen University (KY-2019–016). Written informed consent to participate in this study was provided by the participants' legal guardian/next of kin.

## Author contributions

Conceptualization, visualization, and supervision: X-ZL and CC. Methodology, software, and validation: ZZ and WS. Formal analysis: L-XT. Investigation and resources: RZ, RC, S-NW, and D-MC. Data curation: ZZ, WS, and L-XT. Writing—original draft preparation: ZZ. Writing—review and editing: WS. Project administration: ZZ, WS, X-ZL, and CC. All authors have read and agreed to the published version of the manuscript.
